# Characterization of multifocal T2*-weighted MRI hypointensities in the basal ganglia of elderly, community-dwelling subjects^☆^

**DOI:** 10.1016/j.neuroimage.2013.06.013

**Published:** 2013-11-15

**Authors:** Andreas Glatz, Maria C. Valdés Hernández, Alexander J. Kiker, Mark E. Bastin, Ian J. Deary, Joanna M. Wardlaw

**Affiliations:** a Brain Research Imaging Centre (BRIC), Neuroimaging Sciences, University of Edinburgh, Western General Hospital, Crewe Road, Edinburgh EH4 2XU, UK; b SINAPSE Collaboration, Brain Research Imaging Centre (BRIC), Neuroimaging Sciences, Western General Hospital, Crewe Road, Edinburgh EH4 2XU, UK; c Centre for Cognitive Ageing and Cognitive Epidemiology, University of Edinburgh, Edinburgh, EH8 9JZ, UK; d Department of Psychology, University of Edinburgh, Edinburgh, EH8 9JZ, UK

**Keywords:** Magnetic resonance imaging (MRI), Basal ganglia, Mineralization, Lenticulostriate arterioles, Aging

## Abstract

Multifocal T2*-weighted (T2*w) hypointensities in the basal ganglia, which are believed to arise predominantly from mineralized small vessels and perivascular spaces, have been proposed as a biomarker for cerebral small vessel disease. This study provides baseline data on their appearance on conventional structural MRI for improving and automating current manual segmentation methods. Using a published thresholding method, multifocal T2*w hypointensities were manually segmented from whole brain T2*w volumes acquired from 98 community-dwelling subjects in their early 70s. Connected component analysis was used to derive the average T2*w hypointensity count and load per basal ganglia nucleus, as well as the morphology of their connected components, while nonlinear spatial probability mapping yielded their spatial distribution. T1-weighted (T1w), T2-weighted (T2w) and T2*w intensity distributions of basal ganglia T2*w hypointensities and their appearance on T1w and T2w MRI were investigated to gain further insights into the underlying tissue composition. In 75/98 subjects, on average, 3 T2*w hypointensities with a median total volume per intracranial volume of 50.3 ppm were located in and around the globus pallidus. Individual hypointensities appeared smooth and spherical with a median volume of 12 mm^3^ and median in-plane area of 4 mm^2^. Spatial probability maps suggested an association between T2*w hypointensities and the point of entry of lenticulostriate arterioles into the brain parenchyma. T1w and T2w and especially the T2*w intensity distributions of these hypointensities, which were negatively skewed, were generally not normally distributed indicating an underlying inhomogeneous tissue structure. Globus pallidus T2*w hypointensities tended to appear hypo- and isointense on T1w and T2w MRI, whereas those from other structures appeared iso- and hypointense. This pattern could be explained by an increased mineralization of the globus pallidus. In conclusion, the characteristic spatial distribution and appearance of multifocal basal ganglia T2*w hypointensities in our elderly cohort on structural MRI appear to support the suggested association with mineralized proximal lenticulostriate arterioles and perivascular spaces.

## Highlights

• A rater segmented focal hypointensities on T2*w brain MRI from 98 elderly subjects.
• On average 3 focal hypointensities were found in the basal ganglia of 75 subjects.
• Their spatial distribution suggests an association with lenticulostriate arterioles.
• Signal intensity distributions suggest an underlying inhomogeneous tissue structure.

## Introduction

In the brain, as in the other parts of the body, trace metals, such as iron, are essential for many cellular functions. Iron is specifically needed for dopaminergic neurotransmitter synthesis, myelination of axons and is involved in adenosine triphosphate (ATP) production. Brain iron is present in low-molecular weight complexes, medium-molecular weight complexes, such as transferrin, and high-molecular weight complexes, such as the soluble iron storage protein ferritin and, increasingly with age, the insoluble storage iron-complex hemosiderin. Mineralization of iron and its storage in the form of ferritin are believed to protect the brain from its toxic effects (Mills et al., 2010; Rouault and Cooperman, 2006; Zecca et al., 2004).

Multifocal hypointensities in the basal ganglia are a typical finding on T2*-weighted (T2*w) structural MRI of elderly, otherwise healthy subjects (Penke et al., 2012). They are believed to arise from mineralized tissue predominantly associated with iron encrustations (ferrunginations) and calcifications of lenticulostriate (perforating) arterioles and perivascular spaces (Casanova and Araque, 2003). T2*w hypointensities in the basal ganglia have generally been considered asymptomatic features of aging and only a few studies have documented their chemical composition. Slager and Wagner (1956) analyzed paraffin-embedded autopsy tissue from 200 brains and reported that iron is typically deposited in an organic matrix, which is then followed by the deposition of other trace metals, such as calcium. Morris et al. (1992) visualized iron encrustations around the lenticulostriate arteries of the basal ganglia in frozen samples from 14 brains using Perl's staining method with diaminobenzidin intensification. They reported that iron encrustations did not seem to originate from leaking blood vessels, since they were mostly found in perivascular areas.

Histochemical and chemical methods are still considered the “gold standard” for assessing trace metals in tissue. Brain iron, for example, is commonly visualized with Perl's Prussian Blue tissue stain and can be quantified with the orthophenanthroline method combined with a colorimetrical technique (Hallgren and Sourander, 1958). However, MRI has become the de facto standard for non-invasively visualizing iron and mineral deposits in the brain (Drayer et al., 1986; Schenck and Zimmerman, 2004; Valdés Hernández et al., 2012). In this modality, endogenous magnetic particle deposits accelerate the realignment of water proton spins along the main magnetic field direction as well as their dephasing in the transverse plane. However, their predominant effect depends on their chemical environment (Brass et al., 2006; Schenck and Zimmerman, 2004). This is characterized by a localized shortening of T1, T2 and T2* relaxation times and can lead to focal hyperintensities on T1-weighted (T1w), and hypointensities on T2-weighted (T2w) and T2*w MRI. The T2* relaxation time is defined as 1/T2* = 1/T2 + 1/T2′, where T2′ accounts for additional proton spin dephasing due to gradients in the main magnetic field, magnetic susceptibility differences among tissues, chemical shift effects and imaging gradients applied for spatial encoding (Chavhan et al., 2009). T1 and T2 shortening caused by microscopic magnetic particles is explained by the inner and outer sphere theories, reviewed by Caravan et al. (2009) for example. T2 and T2* shortening due to micro- and mesoscopic particles (Kennan et al., 1994; Weisskoff et al., 1994) depends on the radius *r* of the particle relative to the average proton diffusion coefficient *D* (diffusive correlation time *τ_D_* = *r*^2^/*D*) and its magnetic susceptibility, described by the equatorial Lamor frequency change *δω*. If the particle radius is relatively small (outer sphere regime; *δωτ*_*D*_ ≪ 1) proton spins are irreversibly dephased, which causes a similar shortening of T2 and T2*, and hence hypointensities on T2w and T2*w MRI. If the particle radius is relatively large (static dephasing regime; *δωτ*_*D*_ ≪ 1) protons are reversibly dephased, which causes a shortening of T2* but not T2, and hypointensities on T2*w but not T2w MRI, since proton spins can be fully rephased by the 180° pulse of a spin-echo sequence. A review of the different regimes, including the intermediate regime, can be found in Yung (2003). Notably, flow (Reichenbach et al., 1997) can also cause focal hypointensities on T2*w MRI that mimic iron or mineral deposits. Local changes in tissue composition (Henkelman et al., 1991; Kruer et al., 2012; Valdés Hernández et al., 2012; Vymazal et al., 2000), such as a decrease in the water proton density, can cause hypointensities on T1w MRI.

MRI sequences and post-processing methods for identifying and quantifying iron and mineral stores in the brain were reviewed in Haacke et al. (2005), Ropele et al. (2011) and Valdés Hernández et al. (2012). Novel methods include susceptibility weighted imaging (SWI; Haacke et al., 2009), and quantitative techniques that measure the T2 (Bartzokis et al., 2007), T2* (Aquino et al., 2009; Langkammer et al., 2010) and magnetic susceptibility Δχ (De Rochefort et al., 2010; Schweser et al., 2010) parameters of brain tissue directly. However, daily clinical practice still relies mostly on conventional structural MRI sequences.

In this study, we characterize the appearance of basal ganglia T2*w hypointensities in a group of community-dwelling subjects in their early 70s. This study provides statistics of their count, load and morphological properties per basal ganglia nuclei and their spatial distribution, discusses potential difficulties in their segmentation, and derives their multimodal appearance as a first step towards their application as a possible biomarker for small vessel disease.

## Materials and methods

### Subjects and MRI protocol

T1w, T2w and T2*w whole brain volumes were acquired from the Lothian Birth Cohort 1936 (LBC1936; Deary et al., 2007; Deary et al., 2011; Wardlaw et al., 2011). All participants were imaged using a GE Signa HDxt 1.5 T clinical scanner (Milwaukee, WI, USA) equipped with a self-shielding gradient set (33 mT m^− 1^ maximum gradient strength) and manufacturer supplied 8-channel phased-array head coil; relevant scan parameters are listed in Table 1. The LBC1936 is a longitudinal study of cognitive aging that originally recruited a group of 1091 community-dwelling individuals resident in the Edinburgh and Lothian areas of Scotland who were born in 1936. Approximately three years after they were first recruited into the study, 700 subjects underwent brain MRI at a mean age of 72.5 years (SD 0.7 years). The MRI scans of all participants were categorized according to the General and Putaminal Visual Rating Scale (Valdés Hernández et al., 2011). For our study, a sample was generated containing 100 randomly selected subjects from each category of the General and Putaminal Visual Rating Scale. Two subjects of the sample were excluded due to missing MRI data, which left 98 subjects (45 females) for further analysis.

A representative LBC1936 subject, without major artifacts, significant white matter lesion load or any incidental findings on structural MRI, was chosen as a reference for spatial normalization and intensity standardization. This subject was selected based on head size, brain shape and spatial intensity distribution criteria using the Mahalanobis distance (De Maesschalck et al., 2000).

### Semi-automated segmentation of focal T2*w hypointensities

**Fig. 1:**
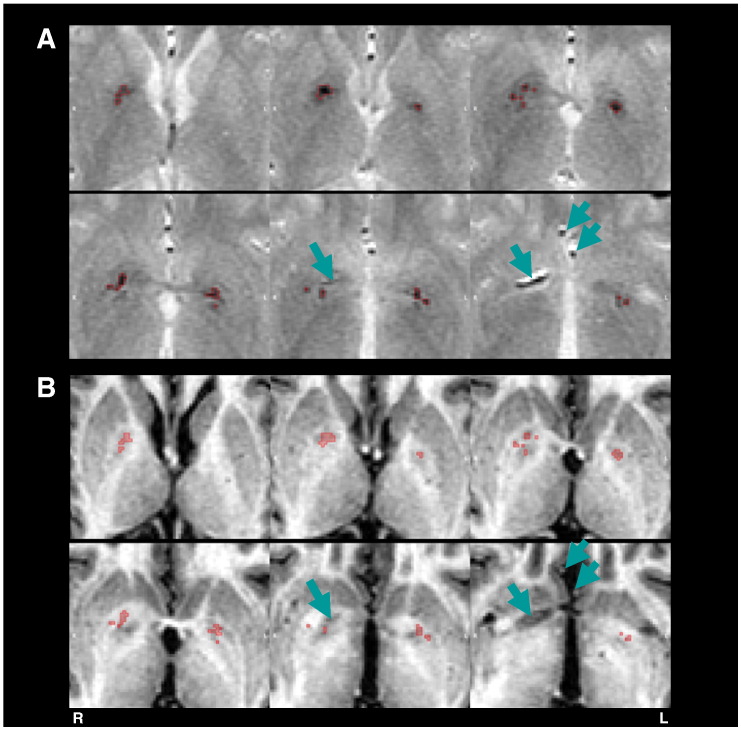
Focal T2*w hypointensities in the basal ganglia of a typical subject. The perimeters of the T2*w hypointensity masks from the rater are indicated by red lines on top of (A) T2*w and (B) co-registered T1w axial slices. This particular subject just exhibits multifocal T2*w hypointensities in the globus pallidus. In more severe cases, T2*w hypointensities are also found in the internal capsule, putamen and caudate. Arrows indicate arterial structures, which mimic T2*w hypointensities on T2*w MRI. However, these features have a branch like appearance and are generally hypointense on T1w MRI.

A trained rater (AJK) used Analyze 10.0 (Mayo Clinic, Rochester, MN, USA) to semi-automatically segment focal hypointensities (Fig. 1) in all brain regions on the T2*w volumes with a local thresholding method described in Valdés Hernández et al. (2011). The rater also delineated regions within the T2*w hypointensities that appeared hypointense on T1w MRI, since they potentially relate to a change in the composition of the underlying tissue (Kruer et al., 2012; Valdés Hernández et al., 2012). The rater segmented 29 subjects twice to estimate the intra-rater variability. The similarity between the T2*w hypointensity masks of the first and second passes was measured using the Jaccard index (Shattuck et al., 2009).

### Preprocessing

#### Co-registration of T1w, T2w and T2*w volumes

All T1w and T2w volumes were affine registered to the corresponding T2*w volumes using FSL FLIRT (Jenkinson et al., 2002) from the FMRIB Software Library (http://fsl.fmrib.ox.ac.uk) with the correlation ratio as the optimization criteria and sinc interpolation with default parameters. In the T2*w volumes, non-brain structures were removed using FSL BET (Smith, 2002) and the obtained brain masks were used to estimate intracranial volume (ICV; Keihaninejad et al., 2010). All non-brain structures in the T1w and T2w volumes were then removed by linearly transforming the brain masks from T2*w to T1w and T2w space and applying them to the corresponding volumes. Non-anatomical intensity variations (bias fields) were finally removed from the brain-extracted T1w, T2w and T2*w volumes with N4 (Tustison et al., 2010).

#### Automated segmentation of basal ganglia nuclei and internal capsule

The basal ganglia nuclei and the thalamus were segmented on the T1w volumes using FSL FIRST (Patenaude et al., 2011) with default parameters and their masks linearly transformed to T2*w space with the corresponding registration matrices (Co-registration of T1w, T2w and T2⁎w volumes section). Internal capsule masks were calculated by dilating the globus pallidus masks with a disk shaped 2D kernel with a radius of 6 mm and subtracting the corresponding thalamus, globus pallidus and caudate masks. The final set of masks required for the subsequent analysis consisted of caudate, putamen, globus pallidus and adjacent internal capsule masks.

#### Automated segmentation of gray/white matter and cerebrospinal fluid

Gray/white matter and cerebrospinal fluid (CSF), which were required for the subsequent intensity standardization, were segmented on co-registered T1w and T2*w volumes using FSL FAST (Zhang et al., 2001) in multi-channel segmentation mode. The a priori tissue class number was set to four to obtain masks selecting gray/white matter, CSF and other voxels. As the volumes were already bias-field corrected, the bias-field correction method of FSL FAST was disabled. Partial volume estimation was enabled to estimate the location of voxels that contained a mixture of tissues (mixels), which were subsequently excluded from the gray/white matter and CSF masks to decrease the random variation of the corresponding intensity histograms. All automatically generated masks were visually checked for major segmentation artifacts, and corrected if necessary.

#### Intensity standardization of T1w, T2w and T2*w volumes

To compare signal intensities of structural T1w, T2w and T2*w volumes across subjects requires their intensities to be standardized (Jager and Hornegger, 2009). A recent method by Hellier (2003) was used for the intensity standardization of all subject volumes, and works by transforming the signal intensities of the subject volumes to maximize the similarity between intensity histograms of the transformed volumes and the histogram of a reference volume. Here, the signal intensities of all T1w, T2w and T2*w volumes were linearly transformed to equalize the robust gray/white matter and CSF intensity means of corresponding volumes from the representative and given subject.

Let *M* ⊂ **Z**^3^ be a set that indexes a three-dimensional *n_x_* × *n_y_* × *n_z_* voxel lattice denoted by

(1) $$M=\left\{i=\left(x,y,z\right)|1<x\leq n_{x},1<x\leq n_{y},1<x\leq n_{z}\right\}\text{.}$$

Then *s*_*i*,*j*,*k*_ is the signal intensity corresponding to voxel *i* ∈ *M*, channel *j* ∈ {T1w,T2w,T2*w} and subject *k* ∈ {1,2,…,*k*^max^}. The linear intensity transformation

(2) $$s_{i,j,k}^{\mathrm{std}}=\alpha_{j,k}s_{i,j,k}+\beta_{j,k}\text{,}$$

of the original T1w, T2w and T2*w signal intensities provides the standardized signal intensities *s*_*i*,*j*,*k*_^*std*^. The coefficients *α*_*j.k*_ and *β*_*j.k*_ were estimated by solving the following linear regression model

(3) $$\bar{S_{j,\mathrm{ref}}^{t}}=\alpha_{j,k}\bar{S_{j,k}^{t}}+\beta_{j,k}+{ɛ}_{j,k}\;\mathrm{with}\;{ɛ}_{j,k}{∼}^{\mathrm{iid}}N\left(0,\sigma_{j,k}^{2}\right)$$

where *S*_*j*,*ref*_^*t*^ = {*s*_*i*,*j*,*ref*_|*i* ∈ *M*_*ref*_^*t*^} and *S*_*j*,*k*_^*t*^ = {*s*_*i*,*j*,*k*_|*i* ∈ *M*_*k*_^*t*^} with *t* ∈ {*GM*,*WM*,*CSF*} are the gray/white matter and CSF signal intensities of the representative and given subject, which were selected by the corresponding masks *M*_*ref*_^*t*^ ⊂ *M* and *M*_*k*_^*t*^ ⊂ *M*. The robust means were estimated with M-estimators of location with the psi-function (LIBRA toolbox; Verboven and Hubert, 2005).

#### Connected component labeling of focal T2*w hypointensities

The locations of individual T2*w hypointensities, i.e. the connected components of the T2*w hypointensity masks from the rater (six-connected neighborhood), that intersected at least 50% with the basal ganglia and internal capsule masks (Automated segmentation of basal ganglia nuclei and internal capsule section) were labeled automatically. The remaining individual T2*w hypointensities were labeled manually and excluded from further processing. T2*w hypointensities that intersected with the internal capsule masks were not excluded because the border between globus pallidus and internal capsule was hard to define and the rater found a marked amount of T2*w hypointensities in this region. All labels were manually checked and corrected if necessary.

Mathematically, the labels were defined as follows. Let *H*_*k*_ = {1,2, …,*h*_*k*_^max^} be the set with the indices of all T2*w hypointensities from the basal ganglia and internal capsule of a subject. The label $l_{h_{k},k}$ of an individual T2*w hypointensity *h*_*k*_ ∈ *H*_*k*_ is then defined by the maximal intersection between its mask $M_{h_{k},k}^{\mathrm{Hypo}}\subset M$ and the structural masks *M*_*l*,*k*_^*BG*^ ⊂ *M*

(4) $$l_{h_{k},k}=\max_{l\in L}\left|M_{h_{k}}^{\mathrm{Hypo}}\cap M_{l,k}^{\mathrm{BG}}\right|\text{,}$$

where *L* = {11,12,13,14,50,51,52,55} denoted the FSL FIRST labels for the left and right basal ganglia nuclei with two additional labels, 14 and 55, for the left and right internal capsules. In the case of an ambiguous maximum, for example if exactly half of the T2*w hypointensity volume extended into two adjacent structures, the structure label that corresponded to the most hypointense part of the T2*w hypointensity was chosen.

### Analysis of basal ganglia T2*w hypointensity masks

T2*w hypointensity masks from the rater were analyzed with a processing pipeline that was mainly implemented in Matlab 2011b (The MathWorks Inc., Natick, MA). The pipeline for generating the spatial distribution map of basal ganglia T2*w hypointensities was implemented in Bash (http://www.gnu.org).

#### Count and load of basal ganglia T2*w hypointensities

The count and load of basal ganglia T2*w hypointensities were derived automatically with the T2*w hypointensity labels (Connected component labeling of focal T2⁎w hypointensities section), and were determined as follows. Let the set with all indices of T2*w hypointensities from a structure be

(5) $$H_{l,k}=\left\{h_{k}|l_{h_{k},k}=l\right\}\text{.}$$

Then the T2*w hypointensity count per structure is

(6) $$n_{l,k}=\left|H_{l,k}\right|\text{,}$$

and the total T2*w hypointensity load *V*_*l*,*k*_^*norm*^ per structure is

(7) $$V_{l,k}^{\mathrm{norm}}=\frac{V_{l,k}}{V_{k}^{\mathrm{ICV}}}=\frac{\left|M_{l,k}^{\mathrm{Hypo}}\right|}{V_{k}^{\mathrm{ICV}}/V^{\mathrm{vox}}}\text{,}$$

where *V*_*l*,*m*_ is the total volume of T2*w hypointensities in a structure, *V*_*k*_^*ICV*^ is the ICV, *V^vox^* is the volume of a single voxel and

(8) $$M_{l,k}^{\mathrm{Hypo}}=\underset{h\in H_{l,k}}{\cup }M_{h,k}^{\mathrm{Hypo}}\text{,}$$

is the mask of all T2*w hypointensities from a structure. The volumes were normalized by ICV since this accounts for the variation of T2*w hypointensity volume with head size (Penke et al., 2012). The two-sided Wilcoxon rank sum test was used to determine the significance of T2*w hypointensity count and load differences in the left and right hemisphere structures.

#### Morphology of individual basal ganglia T2*w hypointensities

Morphological properties of individual T2*w hypointensities that were quantified included their volume, maximum in-plane extent (maximum area), and roundness and sphericity. Roundness and sphericity measures (Wadell, 1932) were originally developed for continuous 3D objects. Therefore similar measures, i.e. the compactness (Bribiesca, 2008) and relative anisotropy, were developed for characterizing discrete 3D objects, and have recently been used to characterize brain microbleeds (Barnes et al., 2011). The compactness and relative anisotropy range from 0 to 1, with perfectly smooth and round discrete 3D objects having a compactness of 1 and relative anisotropy of 0. The morphological properties were calculated with the masks *M*_*h*,*k*_^*Hypo*^ of individual T2*w hypointensities after nearest neighbor interpolation to a 1 mm isotropic voxel lattice.

#### Spatial probability distribution of basal ganglia T2*w hypointensities

The spatial probability distribution of basal ganglia T2*w hypointensities was estimated by linearly aggregating the spatial probability maps of all subjects **p**_*k*_^*spat*^ (Stone, 1961)

(9) $$p^{\mathrm{spat}}=\sum_{k=1}^{k^{\max}}w_{k}p_{k}^{\mathrm{spat}}=\frac{1}{k^{\max}}\sum_{k=1}^{k^{\max}}p_{k}^{\mathrm{spat}}\;\mathrm{with}\;w_{k}=\frac{1}{k^{\max}}\text{.}$$

Non-linear transformation of the T2*w hypointensity mask from a subject

(10) $$M_{k}^{\mathrm{Hypo}}=\underset{h\in H_{k}}{\cup }M_{h,k}^{\mathrm{Hypo}}\text{,}$$

to the T1w reference space of the representative subject (Subjects and MRI protocol section) and normalization yielded the corresponding spatial probability map $p_{k}^{\mathrm{spat}}={\left(p_{1,k}^{\mathrm{spat}},p_{2,k}^{\mathrm{spat}},\ldots ,p_{i^{\max},k}^{\mathrm{spat}}\right)}^{T}$, where *p*_*i*,*k*_^*spat*^ represent the estimated probability densities associated with a reference space voxel. To obtain the warp fields for the non-linear registration, the T1w volume from the representative subject was first rigidly registered to the MNI152 2 mm template using FSL FLIRT (Jenkinson et al., 2002). Then the T2*w volumes were non-linearly registered to the T1w volume from the representative subject using FSL FNIRT (Andersson et al., 2008).

### Analysis of signal intensities selected by focal T2*w hypointensity masks

Segmentation thresholds of basal ganglia T2*w hypointensities were derived to identify factors influencing their segmentation. Then the average T1w, T2w and T2*w intensity probability distributions of these hypointensities and their appearance on T1w and T2w MRI relative to normal-appearing tissue were measured to infer further information about the underlying tissue structure and its composition. This part of the analysis pipeline was fully implemented in Matlab 2011b.

#### Segmentation thresholds of basal ganglia T2*w hypointensities

The segmentation thresholds of the rater *s*_*l*,*k*_^*Thresh*^ were defined as the 97th-percentiles of the standardized T2*w hypointensities from a structure that were selected by the mask *M*_*l*,*k*_^*Hypo*^

(11) $$s_{l,k}^{\mathrm{Thresh}}={\left(S_{l,T2*w,k}^{\mathrm{std},\mathrm{Hypo}}\right)}_{0.97}\;\mathrm{with}\;S_{l,j,k}^{\mathrm{std},\mathrm{Hypo}}=\left\{s_{i,j,k}|i\in M_{l,k}^{\mathrm{Hypo}}\right\}\text{.}$$

The median T2*w intensities of corresponding normal-appearing tissue were used as a reference and given by the median of the standardized T2*w intensities that were selected by the normal-appearing tissue mask *M*_*l*,*k*_^*NABG*^ = *M*_*l*,*k*_^*BG*^ ∩ (*M*_*l*,*k*_^*Hypo*^)^*c*^

(12) $$s_{l,j,k}^{\mathrm{NABG}}={\left(S_{l,j,k}^{\mathrm{std},\mathrm{NABG}}\right)}_{0.5}\;\mathrm{with}\;S_{l,j,k}^{\mathrm{std},\mathrm{NABG}}=\left\{s_{i,j,k}|i\in M_{l,k}^{\mathrm{NABG}}\right\}\text{.}$$

The normal-appearing tissue masks were visually checked and manually corrected if segmentation artifacts were present to ensure a consistent reference across subjects.

#### Signal intensity distributions of basal ganglia T2*w hypointensities

The T1w, T2w and T2*w intensity distributions of basal ganglia T2*w hypointensities were estimated by linearly aggregating the intensity histograms of all subjects **p**_*j*,*k*_^int^ (Stone, 1961)

(13) $$p_{j}^{\mathrm{int}}=\sum_{k=1}^{k^{\max}}w_{k}p_{j,k}^{\mathrm{int}}=\frac{1}{k^{\max}}\sum_{k=1}^{k^{\max}}p_{j,k}^{\mathrm{int}}\;\mathrm{with}\;w_{k}=\frac{1}{k^{\max}}\text{.}$$

The intensity histogram of a subject $p_{j,k}^{\mathrm{int}}={\left(p_{1,j,k}^{\mathrm{int}},p_{2,j,k}^{\mathrm{int}},\ldots ,p_{b_{j}^{\max},j,k}^{\mathrm{int}}\right)}^{T}$, where *p*_*b*,*j*,*k*_^int^ represents the estimated probability density of histogram bin *b*, were calculated with the standardized signal intensities *S*_*j*,*k*_^*std*,*Hypo*^ = {*s*_*i*,*j*,*k*_|*i* ∈ *M*_*k*_^*Hypo*^} and bin widths, which were optimized with all signal intensities *S*_*j*_^*std*,*Hypo*^ = ∪ _∀ *k*_*S*_*j*,*k*_^*std*,*Hypo*^ and the method from Shimazaki and Shinomoto (2007).

The estimated average distributions were characterized in terms of their modality and skewness. The skewness was quantified with the Bowley skewness coefficient (*Q*_*j*_^int,1^ − 2*Q*_*j*_^int,2^ + *Q*_*j*_^int,3^)/*IQR*_*j*_, where the quartiles *Q*_*j*_^int,*q*^ with *q* ∈ {1,2,3,4} and interquartile ranges *IQR*_*j*_ were estimated from **p**_*j*_^int^.

#### Appearance of basal ganglia T2*w hypointensities on T1w and T2w MRI

T2*w hypointensities of a structure with the masks *M*_*l*,*k*_^*Hypo*^ were considered hyper- or hypointense on the T1w and T2w volumes if (i) their signal intensities *S*_*l*,*j*,*k*_^*std*,*Hypo*^ were significantly different from the signal intensities of normal tissue *S*_*l*,*j*,*k*_^*std*,*NABG*^, and (ii) their median signal intensities (*S*_*l*,*j*,*k*_^*std*,*Hypo*^)_0.5_ were brighter or darker than the median signal intensities of normal tissue intensities (*S*_*l*,*j*,*k*_^*std*,*NABG*^)_0.5_. The Mann–Whitney U test was used to assess condition (i) at a significance level of *α* = 0.05.

## Results

### Intra-rater variability and spatial distribution of T2*w hypointensities

The intra-rater variability between 29 T2*w hypointensity masks segmented on two separate occasions was 0.51 ± 0.20, as assessed by the Jaccard similarity index.

As shown in detail in Table 2, the expert rater segmented 867 individual T2*w hypointensities in all subjects, 36% inside and 63% outside the region selected by the basal ganglia and internal capsule masks. Of the 549 individual T2*w hypointensities found outside the basal ganglia and internal capsule, 59% were in the choroid plexus, 10% in the pineal gland, 6% in the substantia nigra and the rest in other structures. Of the 318 individual T2*w hypointensities found inside the basal ganglia and internal capsule, 72% were in the globus pallidus, 18% in the internal capsule, 8% in the putamen and 2% in the caudate.

### Count and load of basal ganglia T2*w hypointensities

**Fig. 2:**
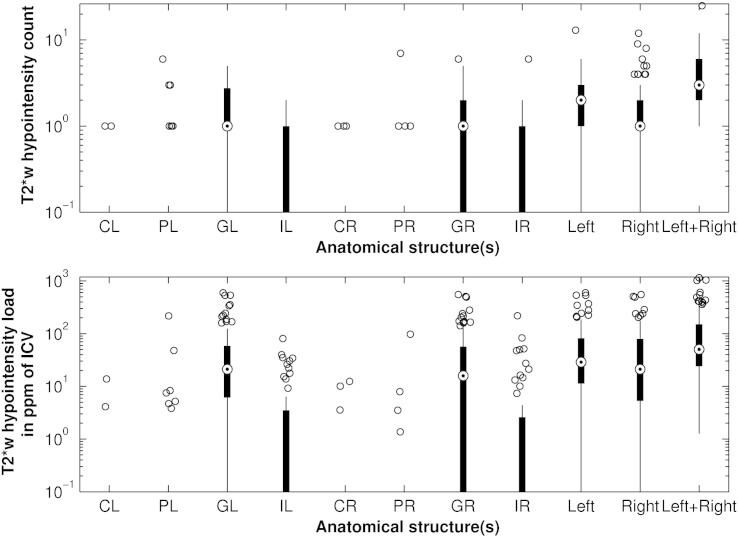
Average count and load of basal ganglia T2*w hypointensities. The anatomical structures include the left caudate (CL), left putamen (PL), left globus pallidus (GL), left internal capsule (IL), right caudate (CR), right putamen (PR), right globus pallidus (GR) and right internal capsule (IR). Additionally, the boxplots show the T2*w hypointensity count in the left and right basal ganglia structures, and in all basal ganglia structures. T2*w hypointensity count and load were especially elevated in the globus pallidus. Although the upper plot suggests a difference between the T2*w hypointensity count in the left and right hemisphere structures, this difference was not significant at *α* = 0.05.

As shown in Fig. 2, each subject with basal ganglia T2*w hypointensities had on average one hypointensity in each globus pallidus, two hypointensities in the left and one in the right hemisphere structures, and 3 hypointensities in all structures. The average hypointensity load in the left and right globus pallidus, in the left and right hemisphere structures, and in all structures was 21.1, 15.9, 28.9, 21.2 and 50.3 ppm, respectively. No significant (*p* < 0.05) differences were found between the number of hypointensities and hypointensity loads in the left and right hemisphere structures, however, Fig. 2 suggests that slightly more hypointensities were counted in the left than right hemisphere structures.

### Morphology of individual basal ganglia T2*w hypointensities

The volume, maximum in-plane area, compactness and relative anisotropy of individual basal ganglia T2*w hypointensities are shown in Table 3. These properties were significantly different for inter-slice hypointensities, which extend across more than one MRI slice, and intra-slice hypointensities, which are contained within one MRI slice. Generally, inter-slice hypointensities were very small, with a smooth surface and a round shape. Due to the limited resolution, intra-slice hypointensities appeared as squares, rectangles and L-shaped elements formed by 1 to 4 voxels.

### Spatial probability distribution of T2*w hypointensities

**Fig. 3:**
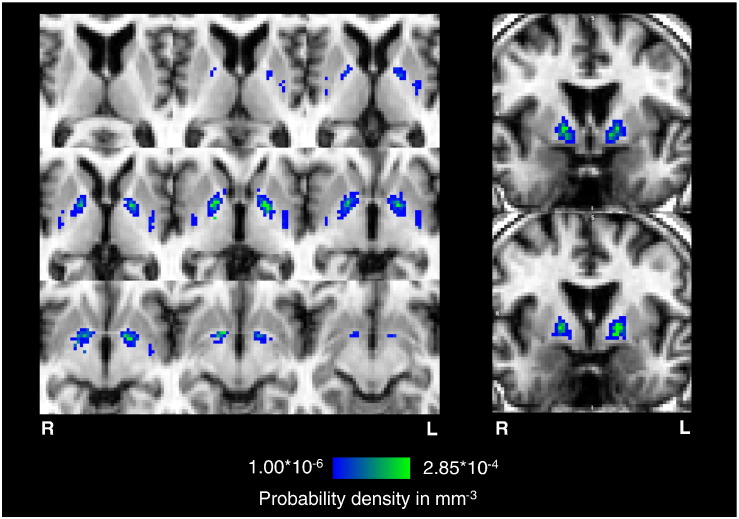
Spatial probability distribution of basal ganglia T2*w hypointensities. The generated spatial distribution maps of T2*w hypointensities are shown overlaid on axial and coronal slices of the 2 mm isotropic T1w reference volume of the representative subject used for non-linear registration. For comparison, the coronal slices approximately correspond to the coronal slices in Feekes et al. (2005), which show the vascular territories of the lenticulostriate arteries (LSA), recurrent arteries of Heubner (RHA), and anterior choroidal arteries (AChA). The spatial probability distribution of these focal T2*w hypointensities indicates a high density inside the globus pallidus (green region), which coincides with the point of entry of the lenticulostriate arterioles into the brain parenchyma; however, their density tends to decrease towards the internal capsule, putamen and caudate.

The spatial distribution map in Fig. 3 indicates a high probability density of T2*w hypointensities within central regions of the globus pallidus in all MRI slices where this structure is evident. From this region the density decreases predominantly not only towards the posterior limb of the internal capsule and putamen, but also the anterior limb of the internal capsule and caudate. T2*w hypointensities also coincide with the posterior putamen. Anatomically, the region with the highest density of T2*w hypointensities coincides with the vascular territories of the lenticulostriate arterioles that supply the globus pallidus (Donzelli et al., 1998; Feekes et al., 2005; Marinkovic et al., 1985).

### Segmentation thresholds of basal ganglia T2* hypointensities

**Fig. 4:**
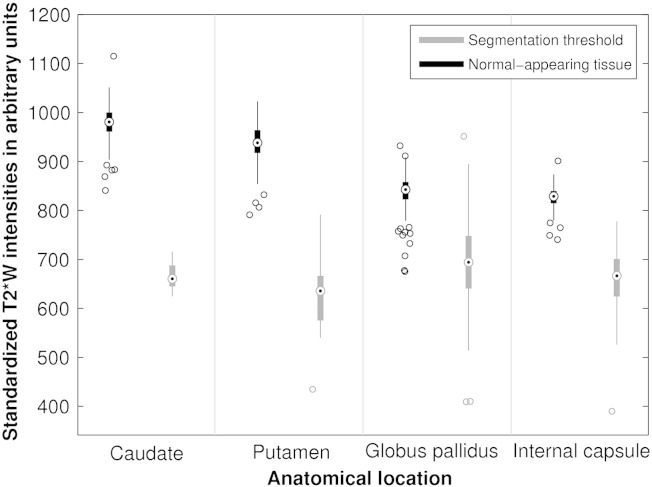
Segmentation thresholds of T2*w hypointensities and median T2*w intensities of corresponding normal-appearing basal ganglia tissue. This figure shows that the rater chose different segmentation thresholds for T2*w hypointensities from different anatomical structures. This systematic variation suggests that the manual segmentation of T2*w hypointensities was potentially influenced by a difference in appearance of T2*w hypointensities from different basal ganglia structures. An additional influence might be the difference in appearance of the normal-appearing tissue intensities of the caudate, putamen, globus pallidus and internal capsule which look increasingly dark on T2*w volumes.

The segmentation thresholds of T2*w hypointensities and the corresponding median signal intensities of normal-appearing tissue, which were similar for the corresponding left and right hemisphere structures, are shown in Fig. 4. The variation in the average segmentation threshold across the different brain structures indicates that the rater adjusted the threshold, which was initially the same for all structures, specifically for each structure. The segmentation thresholds of the caudate, putamen, globus pallidus and adjacent internal capsule were on average 27.3 ± 2.4, 30.0 ± 6.8, 16.5 ± 5.5 and 19.4 ± 5.8% lower than the median signal intensities of normal-appearing tissue, respectively. These values suggest that the rater typically used two different thresholds, one for segmenting T2*w hypointensities in the caudate and putamen, and one for segmenting T2*w hypointensities in the globus pallidus and internal capsule. The normal tissue intensities of the putamen, globus pallidus and adjacent internal capsule were 3.4 ± 2.1, 14.0 ± 2.3 and 15.6 ± 1.7% lower than the normal tissue intensities of the caudate, respectively. These values confirm that the caudate appears brightest on T2*w MRI followed by the putamen, globus pallidus and internal capsule.

### Signal intensity distributions of basal ganglia T2*w hypointensities

**Fig. 5:**
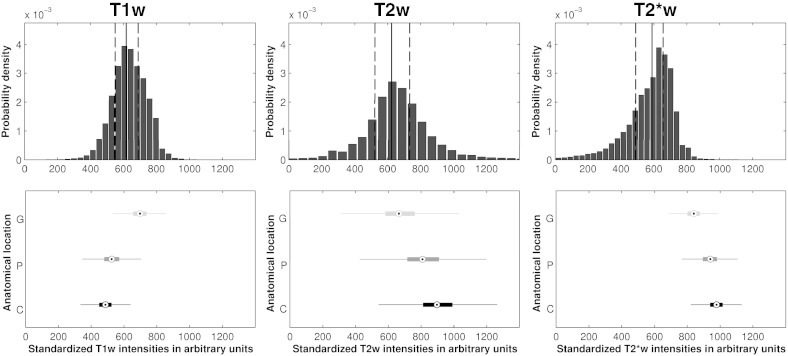
T1w, T2w and T2*w intensity distributions of basal ganglia T2*w hypointensities. The bin-width optimized histograms estimate the distributions of T2*w, T2w and T1w intensities of basal ganglia T2*w hypointensities. The solid vertical lines indicate median and the dashed vertical lines the 25th- and 75th-percentiles. The vertical boxplots of the bottom figures indicate the ranges of normal-appearing intensities of the globus pallidus (G), the putamen (P) and caudate (C). The histograms suggest that the T2*w, T2w and T1w intensity distributions of basal ganglia T2*w hypointensities are unimodal but their shapes generally do not resemble normal distributions and potentially indicate an underlying inhomogeneous tissue structure.

Fig. 5 shows the estimated average T1w, T2w and T2*w intensity distributions of basal ganglia T2*w hypointensities from the cohort. The T1w, T2w and T2*w intensity distributions are all unimodal with Bowley skewness coefficients of 0.044, 0.042 and − 0.193. The skewness coefficients confirm that the T1w and T2w distributions are slightly positively skewed, whereas the T2*w distribution is negatively skewed. These average distribution shapes as well as their skewness indicate that the T1w and T2w distributions, and especially the T2*w distributions are generally not normally distributed. The left tail of the T2*w distributions confirms the observation that there are smaller, darker hypointense regions within individual hypointensities, which are surrounded by larger, brighter hypointense regions.

### Appearance of basal ganglia T2*w hypointensities on T1w and T2w MRI

**Fig. 6:**
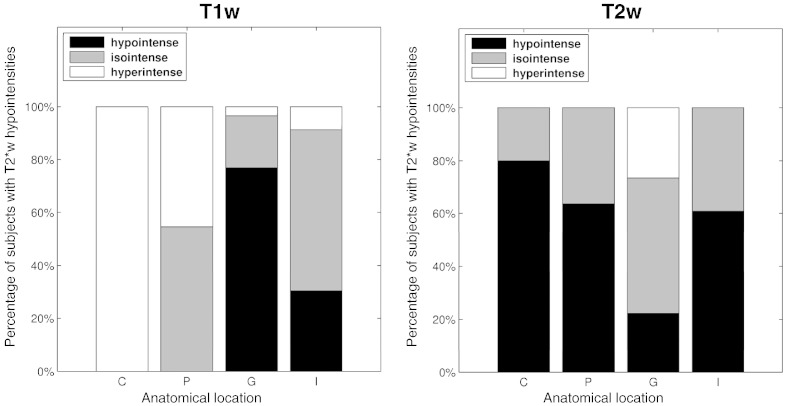
Appearance of basal ganglia T2*w hypointensities on T1w and T2w MRI. This figure shows the percentage of subjects with T2*w hypointensities in a specific basal ganglia or internal capsule region that appeared hypo-, iso- and hyperintense on T1w and T2w MRI. The anatomical structures include the caudate (C), putamen (P), globus pallidus (G) and internal capsule (I). There is a notable difference in appearance on T1w and T2w MRI between T2*w hypointensities in the globus pallidus and other structures. Globus pallidus T2*w hypointensities tend to appear hypo- and isointense on T1w and T2w MRI.

Fig. 6 shows that the appearance of T2*w hypointensities in the globus pallidus on T1w and T2w MRI differs from the appearance of T2*w hypointensities seen in other structures. In 77% of subjects with globus pallidus T2*w hypointensities, these features appeared hypointense on T1w MRI, whereas in 20% they appeared isointense and in 3% hyperintense. Conversely, in 51% of subjects, these globus pallidus T2*w hypointensities appeared isointense on T2w MRI, whereas in 22% they appeared hypointense and in 26% hyperintense. These results are in agreement with the rater (Table 2) who predominantly marked globus pallidus T2*w hypointensities as partly or completely hypointense on T1w MRI. T2*w hypointensities in the internal capsule and putamen appeared isointense on T1w MRI in 61% and 55% of subjects, and hypointense on T2w volumes in 61% and 64% of subjects.

## Discussion

T2*W hypointensities are frequently observed in the basal ganglia of healthy, older subjects, are associated with age-related cognitive decline, and may provide a useful biomarker of cerebral small vessel disease (Penke et al., 2012). These features are believed to arise from mineralization in and around the small lenticulostriate arterioles in the inferior basal ganglia (Casanova and Araque, 2003; Morris et al., 1992). In this study, we document their spatial and intensity distributions, morphology and appearances on T1w and T2w MRI in an older, community dwelling cohort with narrow age range with the aim of improving current manual segmentation methods.

Harder et al. (2008) analyzed focal hypointensities in the globus pallidus and putamen of subjects with a wide age range and found that their SWI signal intensities decreased with age. A rating scheme for counting and classifying hypointensities in the putamen and globus pallidus was also proposed and an increase of the hypointensity grade with age was reported. However, the suggested regularity of the hypointensity pattern and relation with age could not be confirmed in the current study. The former can be, at least partly, attributed to the higher sensitivity of the SWI sequence to iron compared to the T2*w sequence used here (Haacke et al., 2009), and the latter to the very narrow age range of the participants enrolled in this study.

Van Es et al. (2008) investigated hypointensity patterns in the basal ganglia of non-demented elderly subjects and assessed associations between age-related changes in the brain. A method was developed for classifying the hypointensity of the whole caudate, putamen and globus pallidus on T2*w MRI. The study reported that hypointense caudate nuclei are a frequent finding in non-demented elderly subjects and that this change is significantly associated with more atrophy and increased white matter load. However, most of the focal T2*w hypointensities that were found in this study were located in the globus pallidus and far less were observed in the putamen and caudate. This can be attributed to the different analysis methods, variations in subject population as well as differences in imaging parameters, since the TE of the T2*w sequence employed in this study was lower and hence the T2*w volumes were less T2*-weighted (Conijn et al., 2010) than those collected by Van Es et al.

The spatial probability map of basal ganglia T2*w hypointensities (Fig. 3) is consistent with observations in a larger sample of the LBC1936 and shows a high density of T2*w hypointensities at the center of the globus pallidus coinciding with the point of entry of the lenticulostriate arterioles into the basal ganglia (Donzelli et al., 1998; Feekes et al., 2005; Marinkovic et al., 1985). This finding is supported by histological studies (Morris et al., 1992; Slager and Wagner, 1956), which report iron-encrusted vessels in the globus pallidus. Morris et al. (1992) speculated that encrustations make such vessels prone to rupture or cause constriction to the blood flow. Notably, mineralization with a similar spatial distribution pattern as that seen in the T2*w hypointensities studied here was found in the brains of cynomolgus monkeys (Wadsworth et al., 1995). In that study, the authors confirmed that ferrunginations and calcifications were directly related to arterioles of the globus pallidus. Such a relationship could be confirmed in humans in further post-mortem studies where histological findings may be correlated with MRI (Langkammer et al., 2010), or MRI combined with MR angiography, which is able to visualize small vessels in the basal ganglia (Okuchi et al., 2013; Seo et al., 2012).

Brain microbleeds (Cordonnier, 2010) also appear as focal hypointensities on T2*w MRI with a prevalence of 9% in older, otherwise healthy subjects (Cordonnier et al., 2007). Brain microbleeds have maximum diameter between 5 and 10 mm on T2*w volumes and are commonly assumed to be spherical in shape. Barnes et al. (2011) established statistics about their compactness and relative anisotropy. Comparing basal ganglia T2*w hypointensities and brain microbleeds with regard to volume ranges and values of compactness and relative anisotropy suggests that brain microbleeds and hypointensities have a similar appearance and morphology on T2*w MRI although they likely arise due to fundamentally different biological mechanisms and affect different parts of the brain (Charidimou et al., 2012).

The rater typically used different thresholds for segmenting T2*w hypointensities from the caudate and putamen, and globus pallidus and internal capsule. This difference potentially arises from the manual refinement of the initial T2*w hypointensity masks from thresholding (Valdés Hernández et al., 2011), where the rater was likely influence by the appearance of the T2*w hypointensities in the different structures. T2*w hypointensities from the globus pallidus and internal capsule tended to appear uniformly dark with sharper boundaries than T2*w hypointensities from the caudate and putamen, which often appeared more shaded with fuzzier boundaries. Harder et al. (2008) also accounted for this difference in appearance since they proposed alternative classification schemes for the putamen and globus pallidus.

The average T1w, T2w and T2*w intensity distributions of basal ganglia T2*w hypointensities do not resemble normal distributions. In particular, T2*w intensity distributions resemble negatively skewed distributions, such as Beta or reversed Weibull distributions. The finding that T1w, T2w and T2*w signal intensities of MRI features, such as T2*w hypointensities, are not normally distributed is scarcely documented in the MRI literature. For example, current MRI segmentation methods, such as SPM (Statistical Parametric Mapping; http://www.fil.ion.ucl.ac.uk/spm) or FSL FAST, typically model lesions either as a finite mixture of normal distributions (Seghier et al., 2008) or as a uniform distribution (Zhang et al., 2001). Both approaches approximate the signal intensity distributions of hypointense lesions and the segmentation results can deviate significantly from manual segmentations. Alternative data models, such as the Gamma and Beta distributions, have been used by Ho et al. (2002), for example, to model and segment brain tumors on MRI and by Rexilius and Peitgen (2008) to develop a method for the accurate volumetry of small lesions. However, segmentation methods that do not model lesion intensities explicitly but consider them as outliers of the normal-appearing intensity distributions (García-Lorenzo et al., 2011) might produce more accurate results.

The results of the analysis of T1w, T2w and T2*w signal intensities of focal basal ganglia T2*w hypointensities suggest that they might be associated with an inhomogeneous underlying tissue structure, which could be explained by increased mineral deposition. Signal intensities of normal-appearing brain tissue, such as gray and white matter, are generally assumed to be normally distributed (Ashburner and Friston, 2005; Zhang et al., 2001). The fact that T1w, T2w, and especially, T2*w signal intensities of these T2*w hypointensities are generally not normally distributed might be due to irregular changes in tissue structure and composition, such as mineralization in the form of aggregation of trace metals and calcification, as described by Slager and Wagner (1956). These changes can be detected with MRI and could lead to their hypointense appearance on T2*w and T1w MRI (Henkelman et al., 1991; Valdés Hernandéz et al., 2012), as found here in the globus pallidus. Globus pallidus T2*w hypointensities also tend to appear iso- and hypointense on T2w and T2*w MRI. This finding could also be explained by increased mineralization, which could entail increased aggregation of magnetic particles, increased magnetic susceptibility and decreased water proton diffusion around magnetic particles. All these changes (Weisskoff et al., 1994) lead to increased reversible water proton spin dephasing (static dephasing regime) and hence to a more isointense appearance on T2w MRI.

The subjects employed in this study were randomly selected from all participants of the LBC1936, a large longitudinal study of cognitive aging. Because these subjects have a very narrow age rage, this allows individual differences to be identified and analyzed without the major confound of age. The experienced rater was not involved in the selection process and was presented only with the MRI data required to segment T2*w hypointensities with the semi-automated segmentation method described in the Semi-automated segmentation of focal T2⁎w hypointensities section. The masks of the rater were then analyzed independently with the automated methods described above. The study design was therefore chosen to minimize systematic bias, especially from the rating, identification and analysis of the T2*w hypointensities. The results of this study should therefore closely reflect the characteristic appearance of T2*w hypointensities across the whole cohort.

This study has several limitations. Firstly, the T2*w hypointensities were analyzed on gradient-echo magnitude volumes, where the segmentation of T2*w hypointensities was not only complicated by partial volume effects but also blooming artifacts. Focal hypointensities on gradient-echo volumes caused by magnetic particles or complexes generally do not reflect the true particle size because the magnetic field gradients around magnetic particles also cause dephasing and hence hypointensities in voxels adjacent to the particle. For example, studies have shown that brain microbleeds typically appear approximately 1.57 and 2.5 to 5 times bigger in vivo than in post-mortem brains on standard SWI volumes collected from a 3 T scanner and T2*w volumes from a 7 T scanner (De Reuck et al., 2011; Schrag et al., 2010). The apparent size increase of magnetic particles depends on the geometry and magnetic properties of the particle, as well as imaging parameters, such as the echo time (Pintaske et al., 2006). In this study, the echo time of the T2*w sequence (Table 1) was chosen shorter than standard T2*w echo times, which are typically around 40 ms at 1.5 T, to reduce the blooming artifacts at the expense of a decreased sensitivity to magnetic particles (Conijn et al., 2010). A better estimate of the true particle size could be derived from gradient echo phase volumes (McAuley et al., 2011), which were not part of the LBC1936 imaging protocol.

The second limitation is that T2*w hypointensities were just segmented by a single rater, as their segmentation is very time consuming. Therefore no information is available about the exact values of the inter-rater variability. However, the inter-rater variability is expected to be similar to values reported in a previous study, which used the same data for comparing the performance of two manual segmentation methods (Valdés Hernández et al., 2011).

A further limitation is that the bias field correction method N4 (Tustison et al., 2010) was applied to the T1w, T2w and T2*w volumes with the default parameters. N4 is especially suited for bias-field correction on MRI data acquired from elderly subjects, as it does not require a priori information, such as the number of tissue classes. However, it has been suggested (Vovk et al., 2007) that the performance of N4 can potentially be improved by optimizing the input parameters of the algorithm, such as the number of histogram bins.

In conclusion, this study finds that focal T2*w hypointensities in the basal ganglia have a characteristic spatial and signal intensity distribution. Their spatial distribution indicates that they appear to be associated with small vessels, especially those of the globus pallidus, while their signal intensities generally do not resemble normal distributions. These findings as well as their difference in appearance on T1w and T2w MRI further support the hypothesis of mineralized small vessels and perivascular spaces. The evaluation of basal ganglia T2*w hypointensities as a potential biomarker for cerebral small vessel or other vascular disease requires further study in different subject populations ideally with post-mortem data, and is currently limited by a lack of an accurate automated mineral deposit segmentation method.

## Figures and Tables

**Table 1:** Relevant LBC1936 MRI sequences and their parameters. The complete LBC1936 MRI protocol is described in Wardlaw et al. (2011).

Sequence	IR-prep FGRE (3D)	FSE (2D)	GRASS (2D)
Contrast type	T1-weighted	T2-weighted	T2*-weighted
FOV in mm^2^	256 × 256	256 × 256	256 × 256
Orientation	Coronal	Axial	Axial
Slice thickness in mm	1.3	2	2
Acquisition matrix	256 × 256^a^	256 × 256	256 × 256^a^
Flip angle in degrees	8	–	20
TI/TE/TR in ms	500/4/9.8	–/102/11,320	–/15/940
Bandwidth in kHz	15.63	20.83	12.5

a After interpolation by the scanner software.

**Table 2:** Spatial distribution of focal T2*w hypointensities. This table shows the anatomical locations of the individual T2*w hypointensities, i.e. the connected components (six-connected neighborhood) of the T2*w hypointensity masks from the rater. Additionally, it shows how many individual T2*w hypointensities were marked as partly or completely hypointense on T1w MRI. Regions within individual T2*w hypointensities that appeared hypointense on T1w might potentially indicate a change in the underlying tissue composition, such as calcification (Kruer et al., 2012; Valdés Hernández et al., 2012).

Anatomical structure	Total count	Marked as partly hypointense on T1w MRI	Marked as completely hypointense on T1w MRI
*Basal ganglia and internal capsule*			
Caudate	5	0	0
Putamen	26	0	1
Globus pallidus	229	46	5
Internal capsule	58	0	1
Total	318	46	7

*Outside the basal ganglia and internal capsule*			
Choroid plexus	325	4	317
Pineal gland	55	2	52
Substantia nigra	32	0	0
Other structures	137	3	9
Total	549	9	378

**Table 3:** Morphological properties of individual basal ganglia T2*w hypointensities. The properties are significantly different (*p* < 0.05) for inter- (*N_slice_* > 1) and intra-slice (*N_slice_* = 1) basal ganglia T2*w hypointensities.

Type	Inter-slice (*N_slice_* > 1)			Intra-slice (*N_slice_* = 1)			Both (*N_slice_* > = 1)		
Total count	148			170			318		
Percentile	25%	50%	75%	25%	50%	75%	25%	50%	75%
Volume in mm^3^	18	30	106	2	6	8	4	12	30
Maximum area in mm^2^	5	8.5	24.5	1	3	4	2	4	9
Compactness	0.85	0.88	0.91	0.92	0.93	0.95	0.88	0.92	0.93
Relative anisotropy	0.40	0.49	0.59	0.25	0.36	0.57	0.36	0.47	0.57
